# Benefits of pomegranate (*Punica granatum* Linn) fruit extracts to weight changes, total protein, and uric acid in white rats (*Rattus norvegicus*) as an animal model of acute renal failure

**DOI:** 10.14202/vetworld.2016.1269-1274

**Published:** 2016-11-17

**Authors:** Hardany Primarizky, Wiwik Misaco Yuniarti, Bambang Sektiari Lukiswanto

**Affiliations:** Department of Veterinary Clinic, Faculty of Veterinary Medicine, Universitas Airlangga, Surabaya, East Java, Indonesia

**Keywords:** acute renal failure, gout, pomegranate extract, total protein

## Abstract

**Aim:**

The occurrence of acute renal failure (ARF) cases continues to increase every year. Some of the cases are due to nephrotoxic effect caused by overdose of antibiotic consumption or abuse of the drug, gentamicin. An antibiotic therapy that can be used to overcome in such a case is the pomegranate extracts. However, until now, studies using pomegranate for cases of ARF have not been done. This study aims to determine changes in body weight, the levels of total protein (TP), and the levels of serum uric acid (UA) as a result of the pomegranate extract consumption.

**Materials and Methods:**

A total number of 32 rats (*Rattus norvegicus*) were divided into four groups randomly. One group was assigned as the control group (P0) and given intraperitoneal (i.p.) saline and 0.3% carboxy methyl cellulose sodium (CMC) Na; P1 was provided with 80 mg/kg bw/i.p. gentamicin and 0.3% CMC Na orally, P2 was supplied with 80 mg/kg bw/i.p. gentamicin and ellagic acid in 0.3% CMC Na, and P3 was given 80 mg/kg bw/i.p. gentamicin and 150 mg/kg bw pomegranate extract in 0.3% CMC Na. The provision of treatment was carried out in 8 days and followed by making the overthrow of body weight and blood sampling for the examination of study variables.

**Results:**

The results taken by doing the analysis of variance method for the four treatment groups show that the control group (P0) has significant differences from P1, P2, and P3 (p<0.05), but there are no significant differences among the other three treatment groups. Meanwhile, the average values of serum UA levels among P1, P2, and P3 indicate significant differences.

**Conclusion:**

In conclusion, the administration of pomegranate extracts in the treatment of nephrotoxicity toward rats is effective to maintain normal body weight, normal TP levels, and the UA blood serum of the rats. As this study is a preventive therapy, it needs further researches about the effective dose as a curative therapy, its level of effectiveness and its long-term side effects.

## Introduction

Renal failure is the failure of kidneys to remove excess metabolites which are accumulated in the blood. It is a systemic disease and a final common pathway of various urinary tract and kidney diseases [1]. It triggers electrolyte balance disorders, acid-base and water, renal failure. The failure is classified into acute renal failure (ARF) and chronic renal failure [2]. The ARF is characterized by the decrease of the urine volume and an increase of urea and creatinine values in 24 h [3]. The progressive weight loss, increased levels of uric acid (UA), and a decrease of total plasma protein are also major indications of patients with ARF.

The number of patients with kidney failure increases quite a lot and is predicted to continue every year. This is due to factors such as false dose of nephrotoxic drug consumption, lack of public awareness about the dangers of kidney disease and its prevention which should be done from an early age, and so on [4]. Determining kidney damage can be carried out by checking physical parameters such as weight measurement and biochemical parameters such as the examination of the levels of urea, creatinine, UA and the total serum protein.

**Figure-1:**
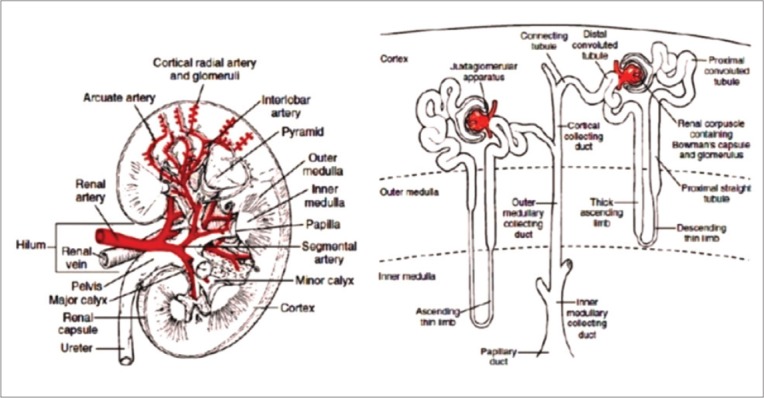
Kidney general structure. (a) The general structure of the kidney, (b) components of the nephron and ductus collectivus system [8].

Kidneys are bean-shaped organs that lie behind the peritoneum, on both sides of the vertebral column. The cross-section of the kidney is divided into two parts, namely, the cortex and the medulla in which the cortex is darker than the medulla [5]. The renal medulla is conical masses which are called renal pyramids [6]. The size of kidney in various species is primarily determined by the number of their nephrons [7]. The general structure of histological kidney can be shown in Figure-1.

Most potassium and UA is reabsorbed by a distal convoluted tubule and secreted into a distal tubule. The formation of ammonia, the acidification of urine, and the water phase setting of the water and acid-base balance ensues in the distal convoluted tubules. The secretion and the selective reabsorption process are completed within the distal convoluted tubules and ductus collectivus [8]. Kidneys have a secretory function, one of which is to excrete end products of nitrogen and protein metabolism (especially urea, UA, and creatinine), foreign chemicals (such as pharmaceuticals), hormones, and other metabolites [9].

**Figure-2:**
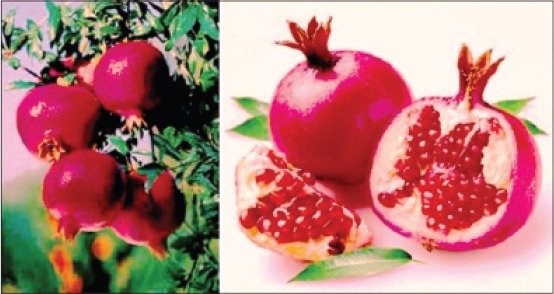
*Punica granatum* L. [11].

Pomegranate fruit is round with the skin colors of green, purple, white, reddish-brown, or purple-black. Its red or white seeds (which are very high in number) are small, slightly flattened, elliptical, and hard [10]. The characteristics of pomegranate can be shown in Figure-2.

One of the components of pomegranate is ellagic acid (EA), an ingredient that can protect cell damage which is caused by free radicals. This capability will synergistically increase when it is combined with another strong antioxidant composition of pomegranate, anthocyanidins [10,12].

The degree of proteinuria and the composition of proteins in the urine depend on the mechanism of kidney injury resulting the loss of protein. A large number of proteins normally passes through the glomerular capillary but does not penetrate the urine. The glomerular wall’s substance and selectivity prevent the transport of albumin, globulin, and protein with other large molecular weight to penetrate its walls. If this barrier is broken, there is a plasma protein leakage in the urine (glomerular proteins). The smaller protein (<20 kDal) is freely filtered but reabsorbed by the proximal tubule. The amount of protein that comes out with urine reduces the levels of the total protein (TP) serum in patients with kidney failures.

UA is a compound of nitrogen produced from catabolism of purines either from diet or endogenous nucleic acids (DNA deoxyribonucleic acid). It is largely excreted through the kidneys and slightly secreted through the gastrointestinal tract. The increasing levels of the UAs called hyperuricemia which can be caused by an excessive production or a decreasing excretion (such as in renal failure). The increasing level of UA in the urine is called uricosuria. The excretion of UA in the urine depends on the levels of UA in the blood, glomerular filtration, and tubular secretion of UA into the urine [13-16].

The results of the previous studies show that 30% of patients who were treated with gentamicin for more than 7 days indicated signs of nephrotoxicity. The gentamicin nephrotoxicity is one of the most common causes of the ARF [17]. Gentamicin belongs to a class of aminoglycosides and is an antibiotic commonly used to treat Gram-negative bacterial infections in humans and animals. It can cause nephrotoxicity and is a way to get an animal model of the ARF. The intraperitoneal (i.p.) administration of gentamicin leads to an increasing formation of superoxide to cause oxidative stress and cellular damage in the proximal renal tubules of the kidney. If it is prolonged, it will cause an ARF [18].

Reactive oxygen species are potential mediators involved in the gentamicin induction of renal impairment. The gentamicin leads to an increasing superoxide anions, peroxynitrite anions, and hydrogen peroxide from the renal cortex mitochondria. A raise of nitric oxide which occurs by the activation of the inducible nitric oxide synthase has proven to cause kidney disorders through several mechanisms. The gentamicin also produces interstitial edema and epithelial necrosis [19].

Currently, there have been many attempts to find cheap and safe alternative medicine for the treatments of the kidney for instance using materials derived from plants. One of the plants whose benefits have been researched is pomegranate. It is a fruit which contains many phenolic compounds, namely, EA and punicalagin. In addition, the fruit is also composed of anthocyanin compounds such as prodelphinidin, delphinidin, sianidin, and pelargonidin [12]. However, until now the use of pomegranate to prevent the ARF has not been done. There are advantages of using herbal medicine such as it is easily produced, its application is simple, and its cost is cheaper than the cost of pharmaceutical drugs. In general, the use of herbal medicine to cure diseases takes a long time, but the effect is to give protection, build and imply positively for other organs. This is different from consuming chemical drugs which have a faster-working process but damage both infected and normal organs [20].

## Materials and Methods

### Ethical approval

This study was duly approved by Institutional Animal Ethics Committee.

### Experimental design

This study is an experimental laboratory research. The research design is a randomized control group - only post-test design. The samples and the treatments were under scalable controlled conditions to keep the effects more valid. The production of animal models and its maintenance were performed in the Laboratory of Biochemistry, Faculty of Medicine, Universitas Airlangga, while the examination of various variables of the study was conducted at the Veterinary Teaching Hospital, Faculty of Veterinary Medicine, Universitas Airlangga. This study was conducted after obtaining a certificate of conduct issued by a research ethics committee.

### Research materials

The experimental units in this study were male strain Wistar white rats (*Rattus norvegicus*) which were obtained from the Animal Care Unit Experiment Universitas Gajah Mada, Yogyakarta. White rats were used because they were inexpensive, easily obtained, and maintained. The rats used in this study should have been male with the criteria of homogeneous samples, the age was 2.5 months old, the weight was between 150 and 200 g, and they were in good health condition, which was characterized by shiny fur sand eyes, and agile movement.

The tools used in this study were 1 cc syringes, a 3-cc syringe and a 10-cc syringe, animal feed and drink containers, husks for the base of the cattle pen, a size 8 feeding tube, a mortar, cotton, tweezers, and a scale. This study used standardized pomegranate extracts which contained 40% EA and pure EA produced by Xi’an Biof Bio-Technology Co., Ltd., gentamicin, 0.3% carboxy methylcellulose (CMC) Na, and 70% alcohol. The samples used were the weight and the 3 ml blood injected out of 32 male rats’ blood which was used for the examination of the TP content and the serum UA.

### Research methods

The standardized pomegranate extracts given to the experimental animals were suspended with 0.3% CMC Na in the mortar to keep the homogeneity of the solvents [21]. The preparations were also made before be given to the experimental animals. The production of 0.3% CMC Na was done by sprinkling 0.3 g CMC Na in 100 ml of warm distilled water.

This study used 32 male rats Wistar aged 2.5 months whose weight was between 150 and 200 g. Having adapted for 1 week, they were divided into four groups, namely, P0, P1, P2, and P3, in which each group was treated in eight cycles. The control group (P0) was given i.p. saline and 0.3% CMC Na orally, P1 was provided with 80 mg/kg bw/i.p. gentamicin and 0.3% oral CMC Na, P2 was parted with 80 mg/kg bw/i.p. gentamicin and 60 mg/kg bw EA in 0.3% CMC Na per oral, and P3 was given 80 mg/kg bw/i.p. gentamicin and pomegranate extracts at a dose of 150 mg/kg bw in 0.3% Na CMC orally. The volume of saline and gentamicin was 0.4 cc, while the volume of the CMC Na solvent, EA and pomegranate extracts was 2 cc. After 8 days of treatment, the weighing and the sampling of intracardiac blood was done. A dose of EA which was administered for 8 days was 60 mg/kg bw/po/day. Based on the content of 40% EA found in the extracts, the fruit extract dose was 150 mg/kg/dd/po/day for 8 days [22]. Weighing had been done before performing blood sampling. The intracardiac blood samples were taken after the white e rats were anesthetized with ether incision in the thoracic region. Blood was collected as much as possible for an examination of the TP content and the serum UA.

The production and checking of the levels of serum TP were conducted by using the Biuret method. Based on the method, the principles of the protein determination levels in serum was the measurement of the purple complex light absorption of proteins to reacting with a biuret reagent. The complex was formed by proteins with Cu^2+^ ions in a biuret reagent under alkaline conditions. The higher the intensity of the absorbed light by the tool meant the higher the protein content was in the serum. The serum UA examination was done by the enzymatic method. The principle of the checking UA levels in enzymatic method was that uricase broke down the UA into allantoin and hydrogen peroxide. Then the presence of peroxidase, peroxide, N-ethyl-N-(2-hidroxy-3-sulfopropy)-3-methylaniline (TOOS) and 4-aminophenazone formed the quinoneimine color. The intensity of the formed red color was proportional with the concentration of UA [23].

The results of the study were presented in a table of the average value and the standard deviation (SD). The treatment effects of the research variables were determined by performing a statistical analysis of variance (ANOVA). They were considered significant if F count > F table or p<0.05. Otherwise, the least significant difference test was proceeded.

## Results

Weighing the white rats was firstly done to examine the variables of the study. The results are shown in Table-1.

**Table 1:** Results of the white rats’ body weight measurement (g) before and after the treatments

Cycles	Weight (g) P0		Weight (g) P1		Weight (g) P2		Weight (g) P3	
			
	Before treatment	After treatment	Before treatment	After treatment	Before treatment	After treatment	Before treatment	After treatment
1	170	195	160	190	175	200	200	200
2	190	200	150	160	170	220	195	220
3	170	190	170	160	155	130	180	180
4	160	190	180	150	170	190	190	205
5	170	175	170	190	190	220	200	240
6	155	175	200	170	150	160	200	220
7	200	225	175	170	180	210	150	150
8	185	215	150	175	200	190	180	180

The research variables are the levels of TP and UA from the blood serum which is taken from all of the rats after 8 days of treatment. The test results of the TP and UA in the Laboratory of Veterinary Teaching Hospital, Faculty of Veterinary Medicine, Universitas Airlangga to 32 rats which were divided into four treatments and eight cycles, namely P0 by administering saline and CMC Na as a control, P1 by administering gentamicin and CMC Na, P2 by administering gentamicin, CMC Na and EA, and P3 by administering gentamicin, CMC Na and pomegranate extract, were processed by applying SPSS 17 for Windows by using ANOVA. The results of blood serum levels of TP and acid veins are shown in Table-2.

**Table 2:** Results of the white rat blood serum examination in the form of TP and UA.

Cycles	P0		P1		P2		P3	
			
	TP	UA	TP	UA	TP	UA	TP	UA
1	6.4	1.5	5.5	1.1	5.5	1.3	5.4	1.2
2	6.2	1.5	6.5	2.7	5.7	1.2	5.3	1.1
3	5.8	1.4	6.2	1.3	5.8	1.1	5.5	1.5
4	6.4	1.8	5.9	1.2	5.6	1.2	6.1	1.2
5	6.9	1.7	5.8	1.2	5.8	1.1	6.0	1.5
6	6.5	1.6	5.7	1.2	7.1	5.7	5.8	1.3
7	6.3	1.2	6.5	1.3	5.9	1.4	5.2	1.1
8	5.7	1.4	5.5	1.6	5.7	1.4	5.6	1.5

TP=Total protein, UA=Uric acid

The average values and the SD of the TP levels in groups P0, P1, P2, and P3 are 6.367±0.3615, 0.3615±5.933, 5.917±0.5913, 5.683±0.3312, respectively. Results of the statistical calculation of the total blood serum proteins are displayed in Table-3.

**Table 3:** Mean and SD of the white rats’ (*R. norvegicus*) total blood serum proteins after treatments.

Treatments	Levels of TP (X̅–±SD)
P0	6.367^a^±0.3615
P1	5.933^b^±0.3615
P2	5.917^b^±0.5913
P3	5.683^b^±0.3312

Different superscripted signs in the same column indicate significant differences P<0.05. *R.* *norvegicus*=*Rattus norvegicus*, SD=Standard deviation, TP=Total protein

The results of the study using ANOVA for the four treatment groups display that the control group (P0) shows a significant difference from the treatment groups (P1, P2, and P3), and there is no significant difference among them. The average values of serum UA levels in groups P0, P1, P2, and P3 are 1.583±0.1472, 1.450±0.6156, 1.8468±1.933, 1.300±0.1673, respectively. The statistical calculation results of the serum UA levels in the blood are shown in Table-4.

**Table 4:** Average values and SD of the serum UA levels in white rat (*R. norvegicus*) after treatments.

Treatments	UA levels (X̅–±SD)
P0	1.583^a^±0.1472
P1	1.450^bc^±0.6156
P2	1.933^c^±1.8468
P3	1.300^b^±0.1673

Different superscripted signs in the same column indicate significant differences P<0.05. *R. norvegicus*=*Rattus* *norvegicus*, SD=Standard deviation, UA=Uric acid

The results reveal that the control group (P0) shows highly significant differences from the treatment groups (P1, P2, and P3), but important distinctions are found among the three groups while a highly important discrepancy is gathered between the treatment groups P2 and P3.

## Discussion

The study of blood serum TPs in white rats between the control group (P0) and the treatment groups (P1, P2, and P3) demonstrates significant differences (p<0.05). P1 displays the highest levels of TPs because this group of white experimental rats with gentamicin was given 0.3% CMC Na. The decreasing levels of TP reflect a decline of the protein amount in the blood due to the gentamicin which has already penetrated into the kidney cells, especially the epithelial cells of the proximal tubule. This causes malfunction, impaired metabolism of the membrane intracellular, and damage of the epithelial cells of proximal tubular kidney which eventually lead to the ARF [24]. The damage of the glomerulus causes the glomerular selectivity walls fail to prevent proteins with large molecular weight to be out of the urine. It induces the leakage of plasma proteins which leads to urine and generates a condition of proteinuria [25].

The serum levels of TPs in white rats by the administration of gentamicin and EA (P2) suffers insignificant reduction than the treatment group P1. It can be noted that there are decreasing levels of the total blood serum proteins which are caused by the improved kidney cells. Meanwhile, the levels of TP blood serum of the white rats by the administration of gentamicin and pomegranate extracts in (P3) sustain inessential degradation than the treatment of group P2. P2 gets the treatment with the best results to reduce the levels of the TPs even though the outcome of the four treatment groups is still within the normal values. The normal levels of the total blood serum proteins of male strain Wistar rats range from 5.0 to 8.0 g/dL [25]. It shows that the combination of various active ingredients in pomegranates has a good effect because it can form a synergistic formulation [26].

Pomegranate (*Punica granatum* Linn) in group P3 contains polyphenols as the main ingredient and punicalagin and EA as the active ingredients. The punicalagin in pomegranates has an antioxidant activity up to 89%. It cannot be directly absorbed by the body because it has a lot of molecules which will undergo hydrolysis in the gut before the absorption. The hydrolysis in the intestine is done by the normal microbes in the gut to become EA which exists in the digestive tract [27]. The low concentrations of EA in the plasma are due to its low solubility in water. The insoluble EA metabolism is caused by microflora activities within the digestive tract. The existence of polyphenols in pomegranate extracts could increase the solubility of EA in the digestive tract. Moreover, polyphenols also have an ability to inhibit the metabolism of EA done by intestinal microflora through their antibacterial activity [12].

The UA levels in the control group (P0) indicate a significant difference from the treatment groups (P1, P2, and P3). The results show that the treatment group P2 gives a better effect in decreasing the UA levels than the treatment group P3. This is because EA contains an antioxidant which decreases the levels of blood biochemistry toward normal values when there is a metabolic disorder in the blood [12]. The normal levels of serum UA blood of male rats strain Wistar range from 0.30 to 1.52 mg/dL [16].

## Conclusion and Suggestion

### Conclusion

The administration of pomegranate extracts as a nephrotoxicity treatment in white rats can maintain a normal weight of the rats, and the normal levels of TP and UA blood serum of white rats.

### Suggestion

As this study is a preventive therapy, it is necessary to conduct further researches on the effective dose of the pomegranate extracts as a curative therapy, the level of effectiveness and their long-term side effects.

## Authors’ Contribution

WMY has planned and designed the work of the research. HP has conducted the research work, such as collecting samples and doing the laboratory work. BSL has analyzed the data and provided technical supports. The article was prepared under the guidance of WMY and BSL. All authors are participated in making of article’s draft, read and approved it.
